# The efficacy of N-acetylcysteine in the management of chronic obstructive pulmonary disease: a systematic review and meta-analysis

**DOI:** 10.7717/peerj.21448

**Published:** 2026-07-16

**Authors:** Xingmin Cai, Sha Peng, Shuixiang Mao, Yong Zhong, Xiubin Yu

**Affiliations:** Department of Respiratory and Critical Care Medicine, Mianzhu People’s Hospital, Deyang, Sichuan, China

**Keywords:** N-acetylcysteine, COPD, Meta-analysis, Chronic obstructive pulmonary disease

## Abstract

**Introduction:**

This study aimed to evaluate the efficacy and safety of N-acetylcysteine (NAC) in patients with chronic obstructive pulmonary disease (COPD).

**Methods:**

This systematic review was registered in PROSPERO (CRD42024597263). In February 2026, a systematic search of PubMed, Embase, Web of Science, and the Cochrane Library was conducted to identify eligible published studies. The methodological quality of the included studies was assessed using the Cochrane Collaboration’s risk-of-bias tool. Statistical analyses, including the generation of forest plots, were performed using Review Manager software.

**Results:**

Fourteen randomized controlled trials involving 2,856 patients were included in the final analysis. The primary analysis of acute exacerbations included 2,609 participants, with 1,295 patients in the NAC group and 1,314 in the control group. Compared with the control group, NAC was associated with a reduced incidence of acute exacerbations in patients with COPD (risk ratio (RR) = 0.87, 95% confidence interval (CI) [0.79–0.96], *P* = 0.006). Subgroup analysis by NAC dosage showed that low-dose NAC (≤600 mg/day) was associated with a lower risk of acute exacerbations (RR = 0.84, 95% CI [0.73–0.97], *P* = 0.02), whereas high-dose NAC (>600 mg/day) showed no statistically significant benefit (RR = 0.88, 95% CI [0.73–1.06], *P* = 0.19). In addition, NAC did not significantly improve pulmonary function, including forced expiratory volume in one second (FEV1) and forced vital capacity (FVC), St. George’s Respiratory Questionnaire scores, or glutathione (GSH) levels. NAC was also not associated with an increased risk of adverse events compared with the control group.

**Conclusions:**

These findings suggest that NAC may reduce the risk of acute exacerbations in patients with COPD without increasing drug-related adverse events. However, the available evidence does not indicate clear benefits for pulmonary ventilation, quality of life, or GSH levels. Further high-quality, large-scale, and rigorously designed randomized controlled trials are needed to confirm these findings.

## Introduction

Chronic obstructive pulmonary disease (COPD) is a progressive respiratory condition characterized by persistent, not fully reversible airflow limitation and an obstructive ventilatory pattern. Disease progression is commonly accompanied by structural changes in the lungs, including persistent inflammation, small-airway narrowing, and destruction of alveolar walls (Christenson et al., 2022). These changes contribute to air trapping, impaired gas exchange, and lung hyperinflation, thereby worsening the clinical course of COPD (Collaborators, 2025; Mathioudakis et al., 2020). COPD is currently the fourth leading cause of death globally, and projections suggest that it may become the third leading cause by 2030 (Iheanacho et al., 2020). Its increasing prevalence, mortality, and economic burden underscore its growing public health impact, particularly in low- and middle-income countries (Vogelmeier et al., 2017). Tobacco smoking remains the primary risk factor for COPD (Yang, Jenkins & Salvi, 2022). In addition to pulmonary manifestations, COPD is frequently associated with systemic complications, such as anemia, osteoporosis, and cardiovascular diseases, which may complicate the management of affected patients (Celli et al., 2004). Current treatment approaches, including inhaled corticosteroids and long-acting bronchodilators, can provide clinical benefits for some patients. However, these therapies have shown variable effects on symptoms or overall respiratory health, and clinically relevant outcomes such as St. George’s Respiratory Questionnaire (SGRQ) scores do not always show consistent improvement (Antoniu, 2009; Calverley et al., 2007). This highlights the urgent need for innovative therapeutic strategies for COPD management (Kim & Criner, 2013).

Mucus hypersecretion is a common symptom in COPD, in which excessive mucus production, combined with impaired clearance, can exacerbate airway obstruction. This further narrows the already compromised airways, making effective mucus clearance and the maintenance of airway sterility important for managing COPD (Kim & Criner, 2013). Mucolytic agents, such as N-acetylcysteine (NAC), dornase-α, and F-actin depolymerizing agents, are commonly used to reduce mucus viscosity by modifying molecular interactions (Kwok et al., 2024). Non-destructive mucolytics, including hypertonic saline and oligosaccharide-based agents, also serve as potential therapeutic options. NAC, derived from the amino acid L-cysteine, is frequently employed because of its mucolytic, antioxidant, and anti-inflammatory properties. Despite its widespread use, the clinical efficacy of NAC remains debated, with studies reporting mixed results on its ability to reduce exacerbations and improve forced expiratory volume in 1 s (FEV1). In addition to mucus retention, the pathogenesis of COPD is closely associated with oxidative stress, primarily triggered by cigarette smoke and environmental pollutants. This oxidative damage may contribute to chronic inflammation, structural lung alterations, impaired lung function, and increased susceptibility to respiratory infections, further complicating COPD management (Singh et al., 2019). NAC, acting as a reactive oxygen species scavenger and a precursor of reduced glutathione, has been primarily studied for preventing exacerbations, while its role in alleviating COPD symptoms remains less well defined. Further investigation and meta-analyses may help clarify the conflicting findings.

In this context, we conducted a systematic review and meta-analysis to assess the impact of NAC on patients with COPD. Our focus was on exacerbations, respiratory symptoms, and quality of life, with the goal of providing high-quality evidence-based guidance for clinical decision-making.

## Methods

### Protocol

This study was conducted in accordance with the Preferred Reporting Items for Systematic Reviews and Meta-Analyses (PRISMA) and the Assessing the Methodological Quality of Systematic Reviews (AMSTAR) guidelines, ensuring standardized reporting standards (Page et al., 2021; Shea et al., 2017). The systematic review was prospectively registered with PROSPERO, an international registry for systematic reviews, under registration ID CRD42024597263.

### Data search strategy

A comprehensive literature search was conducted using the PubMed, Embase, Web of Science, and Cochrane Library databases. The goal was to identify relevant randomized controlled trials on the topic, dating back to the establishment of these databases. The search string used was: ‘(N-acetylcysteine OR Acetylcysteine) AND (chronic obstructive pulmonary disease OR COPD OR pulmonary emphysema OR chronic bronchitis)’ (Table S1). We focused on treatment studies that compared oral NAC with placebo in adult COPD patients. The search included articles published from the earliest available records up to February 20, 2026. Only randomized controlled trials (RCTs) were eligible for inclusion in this meta-analysis. Additionally, previously published systematic reviews and meta-analyses were reviewed to identify any studies that may have been overlooked during the initial search. No language restrictions were applied.

### Study selection and data extraction

To determine the eligibility of the identified trial reports, two authors (XM.C and S.P) independently screened and reviewed each study. Duplicate records were removed first. The titles and abstracts of all retrieved studies were then screened by the two authors to identify potentially eligible studies. After excluding irrelevant studies, the full-texts of the remaining articles were thoroughly reviewed to finalize eligibility decisions.

Data were systematically extracted from eligible full-text articles, including key information such as the author, publication year, and the country where the study was conducted. Details on the study population, including the total number of patients receiving NAC and placebo, NAC dosage, and therapy duration were also collected. Outcome measures included the number of patients without acute exacerbations during the study period, changes in forced expiratory volume in 1 s (FEV1), forced vital capacity (FVC), and St. George’s Respiratory Questionnaire (SGRQ) scores before and after treatment. Additionally, changes in glutathione (GSH) activity and adverse events were extracted to provide an overview of treatment effects and safety profiles. In case of disagreement between the two authors, the issue was resolved through consensus within the review team, with discussions involving a third author (XB.Y) when necessary.

### Inclusion and exclusion criteria

Inclusion criteria were as follows: (1) parallel-group RCTs with placebo controls; (2) adults (≥18 years) with stable COPD (post-bronchodilator FEV1/FVC < 0.70, no acute exacerbation of COPD (AECOPD) in the 4 weeks before enrollment, and no maintenance medication changes in the 2 weeks before randomization); (3) oral NAC (600 mg q24h–1,800 mg q12h) *vs.* matching placebo; (4) reports at least one prespecified outcome. Exclusion criteria included AECOPD patients, non-RCTs/crossover RCTs/preclinical studies, duplicate/inaccessible data, non-oral NAC/combined interventions, follow-up < 4 weeks, or comorbidities (*e.g.*, interstitial lung disease, lung cancer) that may confound outcomes.

### Definitions and outcomes

The primary outcome of this study was the incidence of acute exacerbations during the study period. An acute exacerbation was defined as a worsening of respiratory symptoms (including increased dyspnea, cough, and/or sputum production) in patients with COPD, occurring within 14 days and potentially accompanied by tachypnea and/or tachycardia. The group receiving NAC at a dose of 600 mg per day was classified as the low-dose group, and the group receiving NAC at a dose of 1,200 mg per day was classified as the high-dose group.

Secondary outcomes included changes in forced expiratory volume in 1 s (FEV1) and forced vital capacity (FVC), St. George’s Respiratory Questionnaire (SGRQ) scores, and glutathione (GSH) levels before and after the intervention. The occurrence of adverse events was monitored throughout the study. Key data on adverse events included the risk of study discontinuation due to adverse events, the occurrence of serious adverse events (including those specifically related to COPD), and common side effects such as diarrhea, nausea, headache, and constipation. These comprehensive outcome measures allowed assessment of both efficacy and safety profiles associated with the treatment.

### Assessment of bias

The risk of bias in the included studies was evaluated using the Cochrane Collaboration’s tool for trials, assessing selection, allocation, performance, detection, attrition, and reporting biases. Each study was individually assessed for each bias domain, and the risk of bias was categorized as low, high, or unclear. If sufficient information was not available, the risk of bias was considered unclear. If more than 10 studies were available, contour-enhanced funnel plots were used to explore potential small-study and publication biases, with confidence regions at *p* = 0.05, *p* = 0.025, and *p* = 0.01.

### Data analysis

All statistical analyses were performed using Review Manager version 5.4 (RevMan, The Cochrane Collaboration, Oxford, UK). For dichotomous variables, risk ratio (RR) with 95% confidence intervals (95% CI) was used as the effect size and calculated using the Mantel-Haenszel (M-H) method. A fixed-effects model was used when heterogeneity was low to moderate (I^2^ < 50%), whereas a random-effects model was applied when heterogeneity was substantial (I^2^ ≥ 50%). For continuous variables, the mean difference (MD) was used as the effect size. To evaluate heterogeneity, the *P*-value derived from the chi-square test was used. Additionally, the I^2^ index was employed to summarize heterogeneity. Specifically, a *P*-value less than 0.1 in within-group comparisons or less than or equal to 0.05 in between-group comparisons indicated significant heterogeneity. An I^2^ value of 25% was considered low heterogeneity, 50% moderate, and 75% high heterogeneity, according to established standards. Given the detected heterogeneity, subgroup analysis was conducted based on low-dose and high-dose NAC. Finally, to assess potential publication bias, a visual inspection of the funnel plot was carried out. To minimize type I errors caused by repeated testing or insufficient sample sizes, we performed trial sequential analysis (TSA). TSA evaluates whether the accumulated evidence is sufficient by comparing the cumulative z-curve with predefined conventional and trial sequential monitoring boundaries. The analysis was conducted using TSA software (version 0.9.5.10), with a two-sided test design, an alpha level of 0.05, and statistical power of 80%. The method also calculates the required information size needed to obtain reliable results. Relative risk reduction (RRR) was estimated using data from recent large-scale RCTs as a benchmark. If the cumulative z-curve crosses the TSA boundary, this indicates that the available evidence has reached the required sample size and statistical significance. If the z-curve does not reach the required information size, further data collection may be needed.

## Results

### Description of studies

**Figure 1:**
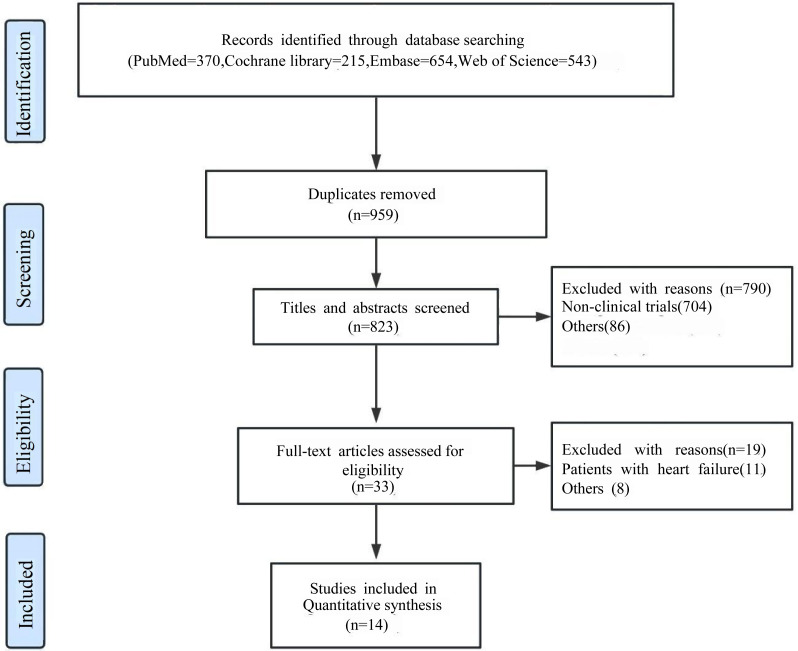
Flow diagram of studies selection process.

A total of 1,782 records were identified through database searching across four databases (PubMed, Cochrane Library, Embase, and Web of Science). After 959 duplicate records were removed, 823 records remained for title and abstract screening. Among these, 790 records were excluded, including 704 non-clinical trials and 86 records excluded for other reasons. The remaining 33 full-text articles were reviewed for eligibility. Following full-text assessment, 19 articles were excluded. Ultimately, 14 randomized controlled trials involving 2,856 patients were included in the meta-analysis (Fig. 1) (Bachh et al., 2007; McGavin et al., 1985; Decramer et al., 2005; Grassi & Morandini, 1976; Hansen et al., 1994; Johnson et al., 2016; Kasielski & Nowak, 2001; Pela et al., 1999; Pirabbasi et al., 2016; Rasmussen & Glennow, 1988; Salve & Atram, 2016; Schermer et al., 2009; Tse et al., 2013; Zheng et al., 2014). The number of participants included in each meta-analysis varied according to outcome availability, as not all studies reported every outcome of interest.

Table 1 presents the baseline data extracted from each of the included studies, including author name, year of publication, drug dose, region, sample size, study design, patient age, proportion of male patients, COPD validation criteria, and duration of follow-up.

### Quality assessment

All included studies were randomized controlled trials, and the quality of the literature was assessed using the Risk of Bias Evaluation Tool for Randomized Controlled Trials (RoB 2.0, Revision 2019). The tool evaluates risk across five domains: bias arising from randomization, bias due to deviations from intended interventions, bias due to missing outcome data, bias in outcome measurement, and bias in selection of the reported result. Each domain was assessed through several signaling questions, and the risk of bias for each domain was categorized as low risk, some concerns, or high risk (Fig. S1).

### Primary outcomes

#### Incidence of acute exacerbations during the study period

**Figure 2:**
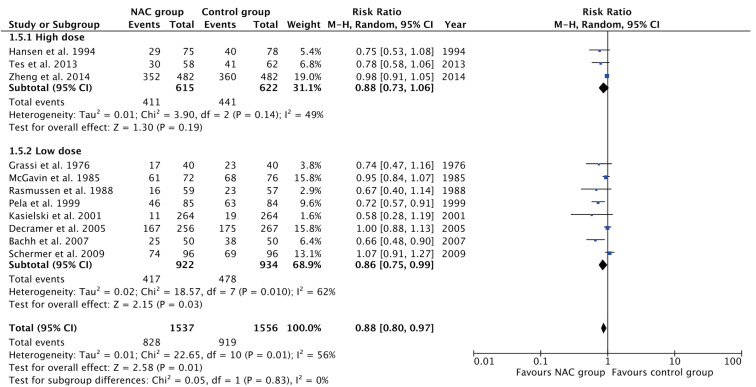
Forest plot and meta-analysis of incidence of acute exacerbations during the study period.

An acute exacerbation was defined as a worsening of respiratory symptoms that required additional therapy. A total of 11 studies reported the number of patients with at least one exacerbation during the study period. In the NAC group, 1,295 patients were included, and in the control group, 1,314 patients were included. The heterogeneity test showed moderate heterogeneity across the included studies (I^2^ = 60%), and the pooled results were therefore analyzed using a random-effects model. Overall, the NAC group had a significantly lower risk of experiencing at least one exacerbation during the study period than the control group (RR = 0.87, 95% CI [0.79–0.96], *P* = 0.006). Subgroup analysis was performed according to NAC dosage, with low dose defined as ≤600 mg/day and high dose defined as >600 mg/day. In the high-dose subgroup, three studies involving 1,237 patients were included. Moderate heterogeneity was observed (I^2^ = 49%), and the point estimate favored NAC, although the difference was not statistically significant (RR = 0.88, 95% CI [0.73–1.06], *P* = 0.19). In the low-dose subgroup, eight studies involving 1,372 patients were included. Heterogeneity was moderate (I^2^ = 66%), and the NAC group showed a significantly lower risk of exacerbation compared with the control group (RR = 0.84, 95% CI [0.73–0.97], *P* = 0.02) (Fig. 2).

**Table 1:** Baseline characteristics of the included trials.

Studies	Countries	Drug dosage	NAC	Control	Study design	Age	Man (%)	Severity of COPD	Followed up
Decramer et al. (2005)	Europe	600 mg qd	256	267	Phase III, two-arm, double-blind, randomized placebo-controlled, parallel-group study	62/62	79/78	Moderate and severe	3 years
Zheng et al. (2014)	China	600 mg bid	504	502	Multicentre, prospective, randomized,double-blind, placebo-controlled, parallel group study	66.2/66.4	82/81	Moderate, severe, and very severe	1 years
Bachh et al. (2007)	India	600 mg qd	50	50	Single-blind,randomized, placebo-controlled study	62.6/60.1	76/80	Moderate and severe	1 years
Schermer et al. (2009)	Netherlands	600 mg qd	96	96	Phase IV, randomized, double blind, double dummy, placebo-controlled study	59.2/59.6	78/67	Mild, moderate, and severe	3 years
Tse et al. (2013)	China	600 mg bid	58	62	Double-blind, randomized, placebo controlled study	71/70.8	93/93	Mild, moderate, severe, and very severe	1 years
Johnson et al. (2016)	USA	1,800 mg bid	23	22	Randomised, double-blind, placebo controlled, parallel	70.1/68.9	86/78	NR	8 weeks
Pirabbasi et al. (2016)	Malaysia	600 mg qd	15	18	A parallel and single blind randomised controlled clinical trial	64/64	NR	NR	6 weeks
Salve & Atram (2016)	India	600 mg qd	47	47	A randomised, parallel group, open labelled, placebo controlled trial	59/58	90/92	Mild, moderate, severe, and very severe	10 weeks
Kasielski & Nowak (2001)	Europe	600 mg qd	22	22	Double-blind,double-dummy comparison between active drug and placebo in two parallel groups	60/60	46/48	Mild and moderate	1 years
Pela et al. (1999)	Europe	600 mg qd	85	84	Open, randomized, controlled study	66/66	80/71	NR	6 months
Hansen et al. (1994)	Europe	1,200 mg qd	75	78	Multicentre, randomized, double-blind, parallel-group study	51.1/51.7	40/46	NR	6 months
McGavin et al. (1985)	Europe	600 mg qd	85	96	Double-blind, placebo-controlled, parallel group study	64.3/62.6	88/83	NR	5 months
Grassi & Morandini (1976)	Europe	600 mg qd	40	40	Multicentre, randomized, double-blind, placebo-controlled study	60/62	65/72.5	NR	6 months
Rasmussen & Glennow (1988)	Europe	600 mg qd	59	57	Double-blind, randomized, placebo controlled study	58.8/58.8	47/38	NR	6 months

### Secondary outcomes

#### Changes in FEV1 volume (liters) before and after the study

**Figure 3:**
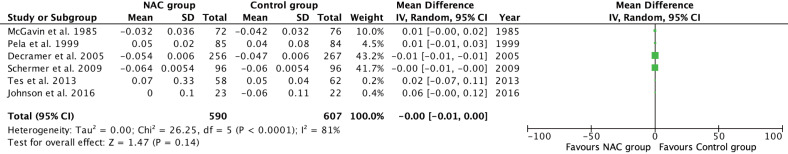
Forest plot and meta-analysis of changes in FEV1 volume in liters before and after the study.

Six studies reported changes in FEV1 volume before and after the study, including a total of 1,197 patients. The results were combined using a random-effects model, accounting for high inter-study heterogeneity (I^2^ = 81%). The analysis showed no statistically significant difference between the NAC and control groups in terms of changes in FEV1 before and after treatment (MD = 0.00, 95% CI [−0.01–0.00], *P* = 0.14) (Fig. 3).

### Changes in FVC volume (liters) before and after the study

**Figure 4:**
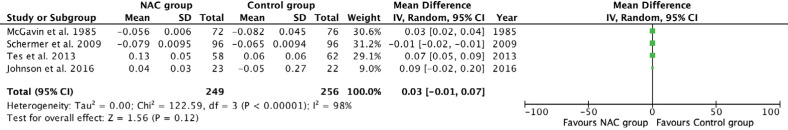
Forest plot and meta-analysis of changes in FVC volume in liters before and after the study.

A total of four studies reported changes in FVC volume before and after the study, with a cumulative total of 505 patients. The results were combined using a random-effects model, considering the high inter-study heterogeneity (I^2^ = 98%). The analysis showed no statistically significant difference between the NAC and control groups in terms of changes in FVC before and after treatment (MD = 0.03, 95% CI [−0.01–0.07], *P* = 0.12) (Fig. 4).

### Occurrence of adverse events

**Figure 5:**
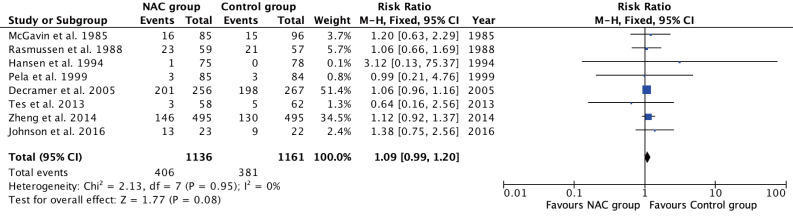
Forest plot and meta-analysis of occurrence of adverse events.

Eight studies reported the occurrence of adverse events, including 2,297 patients. The heterogeneity test suggested homogeneity among the studies (I^2^ = 0%), and the results were combined using a fixed-effects model. The analysis showed no statistically significant difference between the NAC and control groups in the incidence of adverse events (RR = 1.09, 95% CI [0.99–1.20], *P* = 0.08) (Fig. 5).

### St. George’s respiratory questionnaire scores

Three studies reported SGRQ scores before and after treatment, including a total of 259 patients. The heterogeneity test indicated significant heterogeneity among the studies (I^2^ = 94%), and the results were combined using a random-effects model to provide more conservative estimates. The analysis showed no statistically significant difference in SGRQ scores between the NAC and control groups (MD = 1.08, 95% CI [−5.04–7.19]), *P* = 0.73) (Fig. S2).

### GSH level

Two studies reported GSH levels between the NAC and control groups, including a total of 78 patients. Given the homogeneity between studies (I^2^ = 0%), but with only two studies included, a random-effects model was used to combine the results. The analysis indicated that the difference in GSH levels between the NAC and control groups was not statistically significant (MD = 0.14, 95% CI [−0.13–0.40], *P* = 0.32) (Fig. S3).

### Publication bias

Contour-enhanced funnel plots were used to assess potential small-study and publication biases. For the outcome “at least one exacerbation during the study period” a total of 11 studies were included. The funnel plot showed no obvious asymmetry, suggesting no clear evidence of publication bias among the studies (Fig. S4).

### Trial sequential analysis

**Figure 6:**
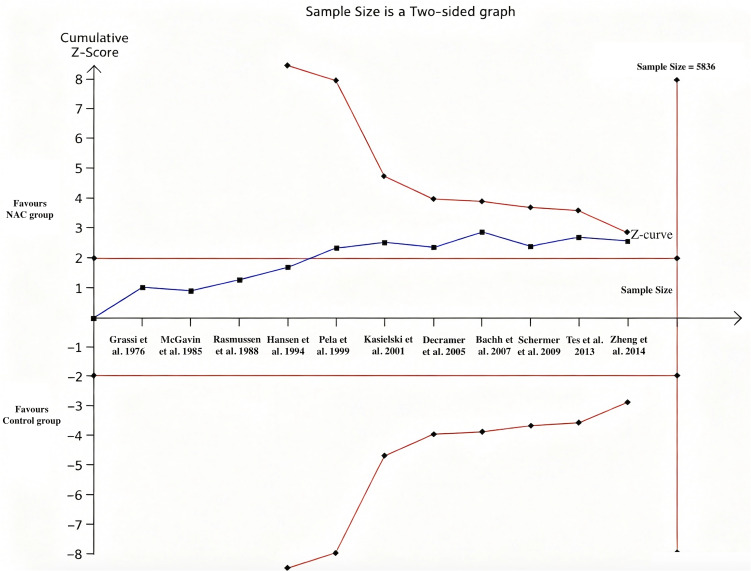
Trial sequential analysis.

The TSA results are presented in Fig. 6. For the primary outcomes, the z-curve crossed the conventional boundary but did not reach the TSA boundary or the required information size. For adverse events, the z-curve did not cross either the conventional boundary or the TSA boundary (Fig. S5). Therefore, additional trials are required, with required information sizes of 5,836 and 11,891, respectively.

## Discussion

COPD is a prevalent chronic lung disorder commonly characterized by symptoms such as cough, dyspnea, and sputum production (Scioscia et al., 2024). NAC has emerged as a potential treatment for managing specific pulmonary traits in COPD patients, owing to its ability to reduce mucus hypersecretion and modulate chronic inflammation and oxidative stress (Scioscia et al., 2024). Although NAC may not be appropriate for all COPD phenotypes, it may provide a useful adjunctive therapy to alleviate respiratory symptoms and reduce exacerbations in a more tailored treatment approach. As a reactive oxygen species scavenger and a precursor of reduced glutathione, NAC has primarily been investigated for its role in preventing disease progression, yet its effectiveness in treating the symptoms of COPD remains uncertain. We performed this meta-analysis to explore the efficacy of N-acetylcysteine in the treatment of chronic obstructive pulmonary disease.

The use of NAC in COPD has been a subject of ongoing debate. Some meta-analyses, which included RCTs, found that NAC did not significantly reduce the risk of acute exacerbations in COPD patients, nor did it improve the decline in lung function (Scioscia et al., 2024). However, other meta-analyses reached different conclusion, suggesting that NAC effectively reduces the incidence of COPD exacerbations, and long-term NAC treatment may decrease the risk of acute exacerbations in COPD (Fowdar et al., 2017; Jiang et al., 2021; Wei et al., 2019). Our study aims to address this controversy and provide clinically relevant evidence for clinical treatment. This is an updated synthesis in this field, with all included studies being of the highest evidence level, RCTs. There are multiple molecular mechanisms of NAC in the treatment of COPD. Patients with COPD are often exposed to substantial oxidative stress due to long-term smoking, chronic inflammation, and environmental pollutants. Glutathione (GSH) is one of the major endogenous antioxidants, and cysteine derived from NAC serves as a rate-limiting substrate for GSH synthesis. Therefore, NAC supplementation may increase GSH levels, enhance antioxidant capacity, and reduce inflammation and tissue injury. In addition, NAC has free sulfhydryl groups, which can directly scavenge free radicals, and this process can also indirectly reduce the degree of activation of inflammatory factors, thus reducing oxidative stress. NAC can also improve the respiratory function of patients with COPD by reducing airway mucus hypersecretion, thereby decreasing sputum viscosity and facilitating sputum clearance. In the process of reducing intracellular oxidative stress, NAC further inhibits the inflammatory response by inhibiting the activation of NF-κB, attenuating the excessive aggregation of neutrophils and macrophages, and can inhibit collagen deposition and airway fibrosis, and promote epithelial protection and repair to ameliorate and reverse the effects of airway remodeling. In clinical practice, however, the extent to which NAC actually benefits COPD patients has been a vexing and controversial issue for clinical experts. As we mentioned earlier, NAC is not necessarily applicable to all COPD patient phenotypes, and our study attempts to further evidence on these controversies based on available RCT data.

Acute exacerbation was defined as an acute worsening of respiratory symptoms that resulted in additional treatment, and the quantitative value of this metric was determined to be the primary outcome of this study. Our study clearly suggests that the incidence of acute exacerbations was significantly lower in patients using NAC than in patients using placebo in a population of 2,609 patients, which supporting the potential effectiveness of using NAC. Our subgroup analysis indicated that low-dose NAC (≤600 mg/day) significantly reduced the risk of COPD exacerbations, while high-dose NAC (>600 mg/day) did not show a clear benefit. This result is consistent with current guideline recommendations that emphasize long-term, low-dose NAC therapy. The lack of effect in the high-dose group may be related to heterogeneity between studies, differences in treatment duration, or reduced adherence with higher pill burden. Overall, our findings suggest that long-term low-dose regimens remain a practical approach supported by the available evidence for COPD patients at risk of exacerbations. Our results are consistent with the results of RCT cohorts in Asian populations, especially in China, but differ from findings in some other populations (Johnson et al., 2016; Schermer et al., 2009; Zheng et al., 2014). We considered the possibility that these inconsistencies might be due to ethnic or COPD phenotypic heterogeneity. However, data limitations prevented further subgroup analyses to explore the differences brought about by the population, which should be addressed in future multicenter studies with larger and more diverse populations.

FEV1 and FVC are commonly used to assess pulmonary ventilatory function and are especially widespread in the diagnosis of COPD and restrictive pneumonia. Our study demonstrated that the use of NAC did not result in a significant improvement in pulmonary ventilation function in patients with COPD, and the conclusions of almost all included studies were consistent with our results.In clinical practice, long-standing pathological changes in COPD may be difficult to reverse with short-term pharmacological treatment alone. Therefore, from the point of view of preventive medicine, pharmacological treatment should be complemented by preventive strategies, including active avoidance of risk factors.

The incidence of drug-related adverse events is an important indicator of drug safety, and our results were consistent with those of the included studies, showing that NAC was not associated with an increased incidence of adverse events in patients with COPD. Similarly, the meta-analysis of SGRQ scores did not demonstrate a clear benefit of NAC, and these results should be interpreted with caution for the following reasons: (1) only three studies reported patients’ SGRQ scores; (2) the high degree of heterogeneity among the included studies reduces the reliability of the results; and (3) the subjective nature of questionnaire-based assessments and differences in administration may have introduced measurement variability. The results of the meta-analysis of changes in GSH should also be interpreted with caution, mainly because of the small number of included studies. Although NAC can indirectly increase GSH concentrations in mechanistic studies, such changes may not be consistently detectable in clinical settings, particularly with complex human environments and limited drug dosages. Therefore, this outcome requires further investigation in future studies.

This study strictly followed all the steps of the PRISMA manual, but there are still some limitations. First, the sample size for some outcomes was limited, which reduces statistical power and may affect the generalizability of the findings. The small sample size of participants, rather than the number of studies, particularly limits the ability to detect meaningful effects in some secondary outcomes. Second, methodological differences among the included studies may have introduced heterogeneity. Third, some included studies were conducted several decades ago, and differences in medical care across time periods may limit the applicability of these findings to current clinical practice.

## Conclusion

In conclusion, our study suggests that NAC use may reduce the risk of acute exacerbations in patients with COPD without increasing drug-related adverse events. In contrast, NAC did not show significant benefits in improving pulmonary ventilation, quality of life, or GSH levels in patients with COPD. Further well-designed, large-sample RCTs are needed to confirm these findings.

##  Supplemental Information

10.7717/peerj.21448/supp-1 Supplemental Information 1 PRISMA checklist

10.7717/peerj.21448/supp-2 Supplemental Information 2 Supplementary documents

10.7717/peerj.21448/supp-3 Supplemental Information 3 Raw Data

## Additional Information and Declarations

### Data Availability

The following information was supplied regarding data availability: This is a Systematic Review/Meta-analysis.
