# Supplementary material for: The efficacy of N-acetylcysteine in the management of chronic obstructive pulmonary disease: a systematic review and meta-analysis
Source: PeerJ. 2026 Jul 16;14:e21448. doi: 10.7717/peerj.21448 (PMC13380882; doi:10.7717/peerj.21448)
Supplement: Supplemental Information 2 [file peerj-14-21448-s002.docx]

**Table S1. Search strategy and results in PubMed database.**

| #1 | "pulmonary disease, chronic obstructive"[MeSH Terms] OR "pulmonary"[All Fields] | 907161 |
| --- | --- | --- |
| #2 | "disease"[All Fields] AND "chronic"[All Fields] AND "obstructive"[All Fields]) OR "chronic obstructive pulmonary disease"[All Fields] OR "copd"[All Fields] OR ("pulmonary disease, chronic obstructive"[MeSH Terms] OR "pulmonary"[All Fields] AND "disease"[All Fields] AND "chronic"[All Fields] | 141843 |
| #3 | "obstructive"[All Fields] OR "chronic obstructive pulmonary disease"[All Fields] OR "chronic"[All Fields] | 1842845 |
| #4 | "obstructive"[All Fields] AND "pulmonary"[All Fields] AND "disease"[All Fields] OR ("bronchitis, chronic"[MeSH Terms] OR "bronchitis"[All Fields] | 125789 |
| #5 | "chronic"[All Fields] OR "chronic bronchitis"[All Fields] OR ("chronic"[All Fields] AND "bronchitis"[All Fields] | 17525 |
| #6 | "acetylcystein"[All Fields] OR "acetylcysteine"[MeSH Terms] OR "acetylcysteine"[All Fields] OR ("acetylcysteine"[MeSH Terms] OR "acetylcysteine"[All Fields] OR "n acetylcysteine"[All Fields]) OR "NAC"[All Fields] | 46956 |
| #7 | #1 ADN #2 AND #3 AND #4 AND #5 AND #6 | 316 |


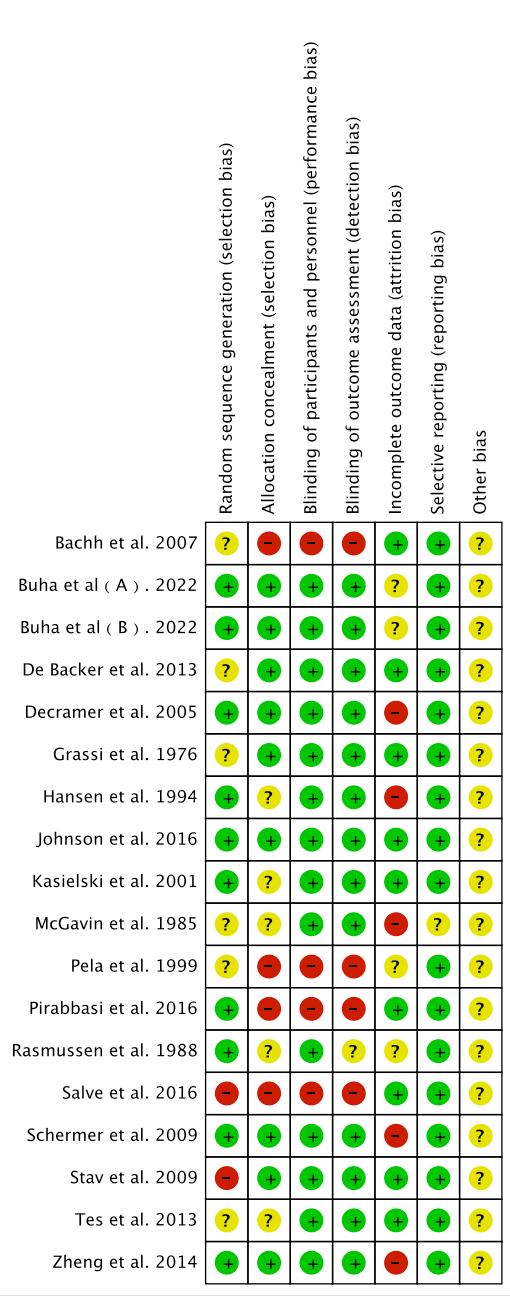


**Figure S1. The results of Risk of bias.**


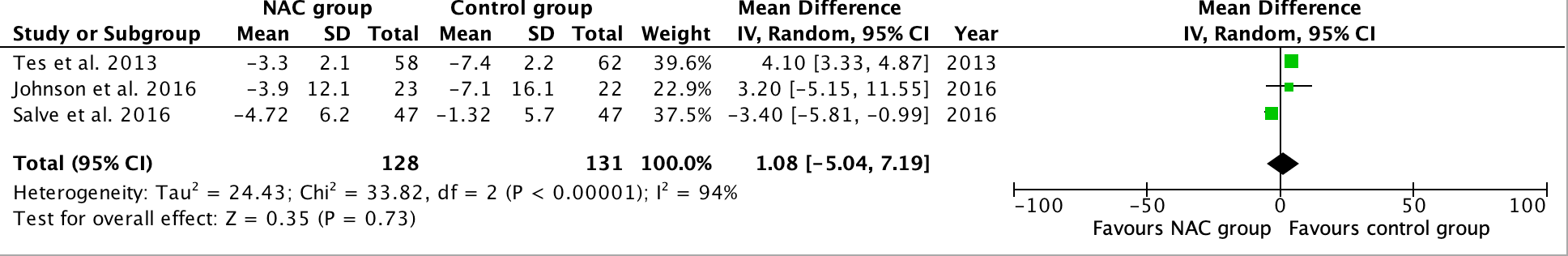


Figure S2. Forest plots for SGRQ score between the N-acetylcysteine therapy group and the control group.


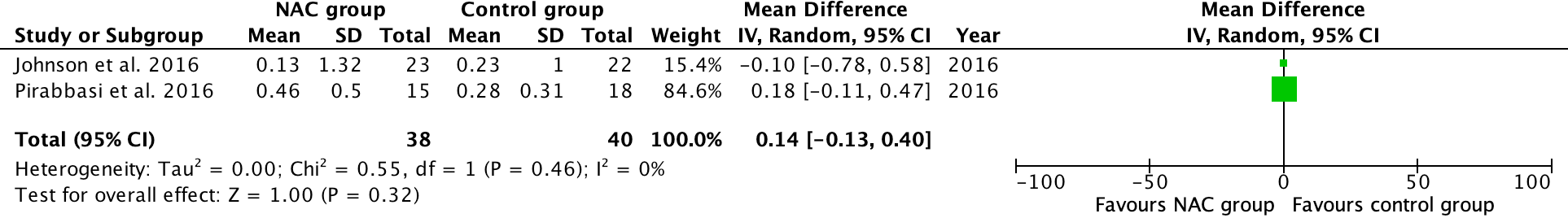


Figure S3. Forest plots for GSH level (µM) between the N-acetylcysteine therapy group and the control group.

A B


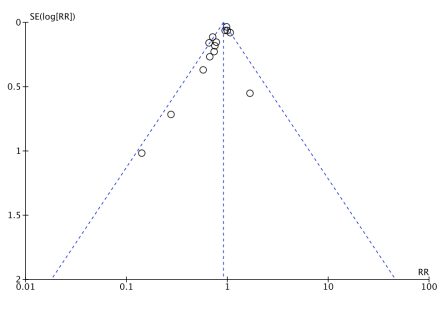

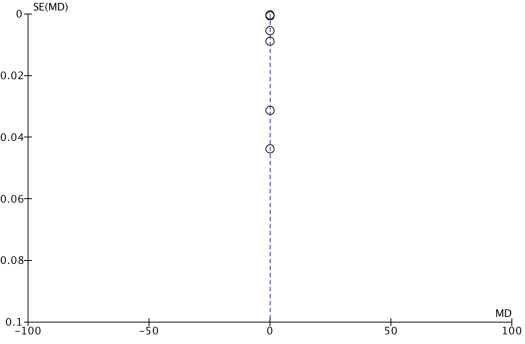


C D


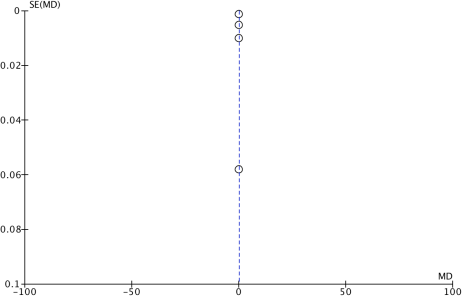

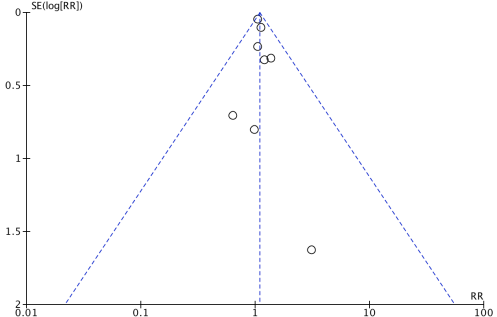


**Figure S4. funnel plots.**

1. The incidence of acute exacerbations; B.The change in FEV1; C.The change in FVC; D.Adverse events.


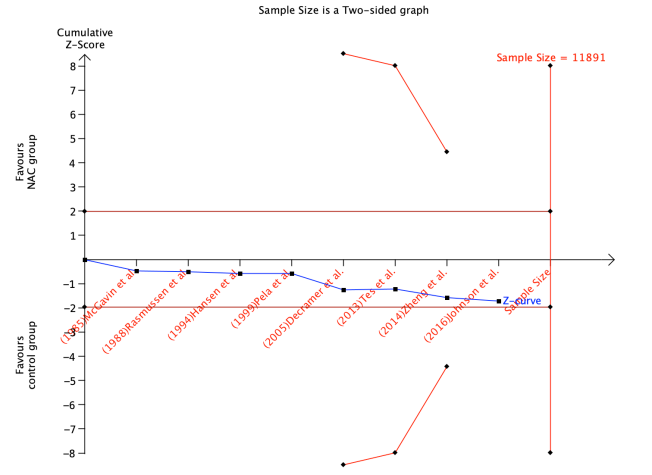


**Figure S5. The TSA of adverse events.**
