# Supplementary material for: The efficacy of N-acetylcysteine in the management of chronic obstructive pulmonary disease: a systematic review and meta-analysis
Source: PeerJ. 2026 Jul 16;14:e21448. doi: 10.7717/peerj.21448 (PMC13380882; doi:10.7717/peerj.21448)
Supplement: Supplemental Information 3 [file peerj-14-21448-s003.docx]

• **Decramer M, Rutten-van Mölken M, Dekhuijzen PN, Troosters T, van Herwaarden C, Pellegrino R, et al. Effects of N-acetylcysteine on outcomes in chronic obstructive pulmonary disease (Bronchitis Randomized on NAC Cost-Utility Study, BRONCUS): a randomised placebo-controlled trial. Lancet (London, England). 2005;365(9470):1552-60.**
DOI: 10.1016/S0140-6736(05)66456-2

• • **Zheng JP, Wen FQ, Bai CX, Wan HY, Kang J, Chen P, et al. Twice daily N-acetylcysteine 600 mg for exacerbations of chronic obstructive pulmonary disease (PANTHEON): a randomised, double-blind placebo-controlled trial. The Lancet Respiratory medicine. 2014;2(3):187-94.**
DOI: 10.1016/S2213-2600(14)70034-5

• • **Bachh AA, Shah N, Bhargava R, Ahmed Z, Pandey D, Dar K. Effect of oral N-acetylcysteine in COPD–a randomised controlled trial. JK Practitioner. 2007;14(1):12-6.**
DOI: 10.1016/j.jkpract.2007.01.005

• • **Schermer T, Chavannes N, Dekhuijzen R, Wouters E, Muris J, Akkermans R, et al. Fluticasone and N-acetylcysteine in primary care patients with COPD or chronic bronchitis. Respiratory medicine. 2009;103(4):542-51.**
DOI: 10.1016/j.rmed.2008.09.015

• • **Stav D, Raz M. Effect of N-acetylcysteine on air trapping in COPD: a randomized placebo-controlled study. Chest. 2009;136(2):381-6.**
DOI: 10.1378/chest.08-3022

• • **Tse HN, Raiteri L, Wong KY, Yee KS, Ng LY, Wai KY, et al. High-dose N-acetylcysteine in stable COPD: the 1-year, double-blind, randomized, placebo-controlled HIACE study. Chest. 2013;144(1):106-18.**
DOI: 10.1378/chest.12-2357

• • **Johnson K, McEvoy CE, Naqvi S, Wendt C, Reilkoff RA, Kunisaki KM, et al. High-dose oral N-acetylcysteine fails to improve respiratory health status in patients with chronic obstructive pulmonary disease and chronic bronchitis: a randomized, placebo-controlled trial. International journal of chronic obstructive pulmonary disease. 2016;11:799-807.**
DOI: 10.2147/COPD.S102375

• • **Pirabbasi E, Shahar S, Manaf ZA, Rajab NF, Manap RA. Efficacy of Ascorbic Acid (Vitamin C) and/N-Acetylcysteine (NAC) Supplementation on Nutritional and Antioxidant Status of Male Chronic Obstructive Pulmonary Disease (COPD) Patients. Journal of nutritional science and vitaminology. 2016;62(1):54-61.**
DOI: 10.3177/jnsv.62.54

• • **Salve VT, Atram JS. N-Acetylcysteine Combined with Home Based Physical Activity: Effect on Health Related Quality of Life in Stable COPD Patients- A Randomised Controlled Trial. Journal of clinical and diagnostic research : JCDR. 2016;10(12):Oc16-oc9.**
DOI: 10.7860/JCDR/2016/21195.8984

• • **Pela R, Calcagni AM, Subiaco S, Isidori P, Tubaldi A, Sanguinetti CM. N-acetylcysteine reduces the exacerbation rate in patients with moderate to severe COPD. Respiration; international review of thoracic diseases. 1999;66(6):495-500.**
DOI: 10.1159/000029740

• • **Hansen NC, Skriver A, Brorsen-Riis L, Balsløv S, Evald T, Maltbaek N, et al. Orally administered N-acetylcysteine may improve general well-being in patients with mild chronic bronchitis. Respiratory medicine. 1994;88(7):531-5.**
DOI: 10.1016/S0954-6111(05)80174-2

• • **McGavin CR, Macfarlane JT, Prescott RJ, Elmes P, Ferguson A, Nariman S, et al. Oral N-acetylcysteine and exacerbation rates in patients with chronic bronchitis and severe airways obstruction. Thorax. 1985;40(11):832-5.**
DOI: 10.1136/thx.40.11.832

• • **Grassi C, Morandini GC. A controlled trial of intermittent oral acetylcysteine in the long-term treatment of chronic bronchitis. European journal of clinical pharmacology. 1976;09(5-6):393-6.**
DOI: 10.1007/BF00614132

• • **Rasmussen JB, Glennow C. Reduction in days of illness after long-term treatment with N-acetylcysteine controlled-release tablets in patients with chronic bronchitis. The European respiratory journal. 1988;1(4):351-5.**
DOI: 10.1183/09031936.88.01040351

• • **Kasielski M, Nowak D. Long-term administration of N-acetylcysteine decreases hydrogen peroxide exhalation in subjects with chronic obstructive pulmonary disease. Respiratory medicine. 2001;95(6):448-56.**
DOI: 10.1053/rmed.2001.1066

•
